# *TINF2* Gene Mutation in a Patient with Pulmonary Fibrosis

**DOI:** 10.1155/2016/1310862

**Published:** 2016-03-20

**Authors:** T. W. Hoffman, J. J. van der Vis, M. F. M. van Oosterhout, H. W. van Es, D. A. van Kessel, J. C. Grutters, C. H. M. van Moorsel

**Affiliations:** ^1^Department of Pulmonology, St. Antonius Hospital, Koekoekslaan 1, 3435 CM Nieuwegein, Netherlands; ^2^Department of Clinical Chemistry, St. Antonius Hospital, Koekoekslaan 1, 3435 CM Nieuwegein, Netherlands; ^3^Department of Pathology, St. Antonius Hospital, Koekoekslaan 1, 3435 CM Nieuwegein, Netherlands; ^4^Department of Radiology, St. Antonius Hospital, Koekoekslaan 1, 3435 CM Nieuwegein, Netherlands; ^5^Division of Heart and Lungs, University Medical Center, Heidelberglaan 100, 3584 CX Utrecht, Netherlands

## Abstract

Pulmonary fibrosis is a frequent manifestation of telomere syndromes. Telomere gene mutations are found in up to 25% and 3% of patients with familial disease and sporadic disease, respectively. The telomere gene* TINF2* encodes an eponymous protein that is part of the shelterin complex, a complex involved in telomere protection and maintenance. A* TINF2* gene mutation was recently reported in a family with pulmonary fibrosis. We identified a heterozygous Ser245Tyr mutation in the* TINF2* gene of previously healthy female patient that presented with progressive cough due to pulmonary fibrosis as well as panhypogammaglobulinemia at age 52. Retrospective multidisciplinary evaluation classified her as a case of possible idiopathic pulmonary fibrosis. Telomere length-measurement indicated normal telomere length in the peripheral blood compartment. This is the first report of a TINF2 mutation in a patient with sporadic pulmonary fibrosis, which represents another association between* TINF2* mutations and this disease. Furthermore, this case underlines the importance of telomere dysfunction and not telomere length alone in telomere syndromes and draws attention to hypogammaglobulinemia as a manifestation of telomere syndromes.

## 1. Introduction

Idiopathic pulmonary fibrosis (IPF) has been described as a condition of “accelerated aging” of the lungs and the prevalence of IPF rises dramatically with age [1]. IPF is characterized by progressive fibrosis, leading to respiratory failure and eventually death. The pathogenetic processes leading to IPF are still not completely understood, but in a subset of patients telomere dysfunction plays a key role [2].

Telomere dysfunction is the hallmark of the telomere syndromes, conditions that are clinically characterized by premature aging and are exemplified by dyskeratosis congenita (DC) [3]. Telomeres are repetitive TTAGGG sequences at chromosome ends that serve as a solution to the end-replication problem and end-protection problem that arise in cells with linear DNA [4]. Every cell division results in shorter telomeres, and with increasing age telomeres become critically short and induce cellular senescence or apoptosis [5]. Telomeres are highly regulated structures that are maintained by various regulatory proteins. Mutations have been found in genes encoding parts of the telomerase and the shelterin complex, as well as other genes involved in telomere biology [6]. In IPF patients, mutations have been found in* TERT*,* TERC*,* DKC1*,* RTEL1*, and* PARN* genes [7–11].

Mutations in telomere genes can lead to critically short telomeres in both high-turnover and low-turnover tissues, resulting in various disease phenotypes [3]. In the lung, a slow-turnover tissue, exogenous damage in combination with short telomeres due to telomere gene mutations, is suggested to trigger pulmonary fibrosis [3]. Mutations in telomere genes have been found in up to 25% of familial and 1–3% of sporadic IPF patients [3, 9–11]. In fact, although DC was the first telomere syndrome to be identified, it is now understood that IPF is by far the most common manifestation of a telomere syndrome [12].

**Figure 1:**
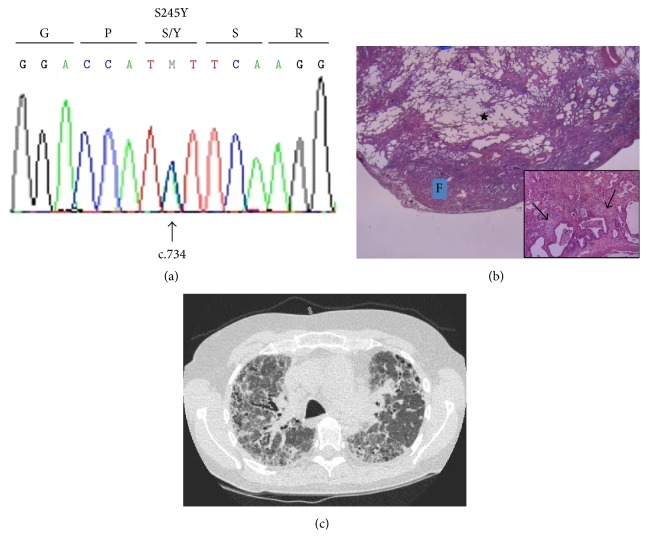
(a) DNA sequence of a segment of* TINF2* exon 6 demonstrates a cytosine to adenine change at position c.734 that leads to the amino acid substitution of serine to tyrosine at codon 245. M denotes that both a cytosine and an adenine nucleotide at cDNA position 734 are present, indicating a heterozygous mutation. (b) Lung biopsy specimen of our patient taken at the time of diagnosis (H&E 12,5x). The biopsy shows temporal and spatial heterogeneous fibrosis consistent with a usual interstitial pneumonia (UIP) pattern: marked subpleural fibrosis with honeycombing (F) and central sparing (★), and the presence of fibroblast foci (inset 200x, arrows). No features suggestive of an alternative diagnosis were seen. Specifically, histologically, there was no granulomatous disease or lymphocytic interstitial pneumonia pattern present suggestive of granulomatous-lymphocytic interstitial lung disease (GLILD) and there was no interstitial elastosis suggestive of pleuroparenchymal fibroelastosis (PPFE). (c) HRCT scan image of the lungs of our patient when she was referred for lung transplantation. The scan shows thickening of the inter- and intralobular septae, in both the subpleural and peribronchovascular areas. Honeycombing is seen on the left. This is inconsistent with a UIP pattern, due to the peribronchovascular extension of the fibrosis. No radiological features suggestive of alternative diagnoses were seen. Specifically, there were no pulmonary micronodules that are typical of GLILD, and there was no pleuroparenchymal thickening in the upper lung zones, which is typical of PPFE. With these findings combined, the patient can be classified as a case of possible IPF, in accordance with current guidelines [14].

In addition to the previously mentioned telomere genes, a mutation has recently been reported in the TRF1-Interacting Nuclear Factor 2 gene (*TINF2*) in the setting of familial pulmonary fibrosis [13]. The* TINF2* gene encodes an eponymous protein, which is part of the shelterin complex.* TINF2* mutations have often been found to underlie telomere syndrome manifestations other than pulmonary fibrosis [6]. Therefore, we performed a sequence analysis of* TINF2* in our cohort of 158 pulmonary fibrosis patients, which revealed a heterozygous Ser245Tyr mutation in one patient (Figure 1). All patients provided formal written consent.

## 2. Case Presentation

The patient was a 52-year-old female that presented with progressive cough and dyspnea for 8 weeks. Her medical history was unremarkable; she did not suffer from chronic disease. She had never smoked and had no known allergies or exposure to toxics. She had no family history suggestive of telomere syndromes or lung disease and she had two healthy daughters. She was admitted to a general hospital, where she was diagnosed with IPF based on high-resolution computed tomography scan and open lung biopsy findings. Simultaneously, laboratory analysis revealed that she had severe panhypogammaglobulinemia (IgM 0.32 g/L; IgA < 0.1 g/L; and IgG 2.2 g/L), and she was given the diagnosis common variable immunodeficiency (CVID). There were no clinical features suggestive of telomere disease or autoimmune disease. The patient had no history of recurrent infections. Bronchoalveolar lavage fluid showed no lymphocytosis or other abnormalities, and antinuclear antibody and anti-neutrophil cytoplasmic antibody tests were negative. She was started on oral corticosteroid treatment for her pulmonary fibrosis (as was common practice at the time) and gammaglobulin replacement therapy, both of which she would receive during her entire course of disease.

Her situation was relatively stable for 48 months, and she showed no signs of infections. When she became progressively dyspnoic, she was screened for lung transplantation. Because there were no contraindications for lung transplantation, she was placed on the waiting list 65 months after diagnosis. Unfortunately, a donor was not available in time and she passed away 71 months after diagnosis at the age of 58. As this all occurred a decade ago, we have retrospectively reviewed the case based on current guidelines [14] in our multidisciplinary interstitial lung diseases team. Based on pathological and radiological findings, and in the absence of features suggestive of other diagnoses, our patient can be classified as a case of possible IPF (Figure 1). There was multidisciplinary consensus on the clinical diagnosis of IPF, additionally supported by the progressive nature of the fibrosis in this patient and the failed response to corticosteroid treatment. We retrospectively measured the T/S ratio in DNA extracted from peripheral blood monocytes obtained 65 months after diagnosis and found a T/S ratio of 1.03, indicating normal telomere length in this cell compartment.

## 3. Discussion

**Figure 2:**
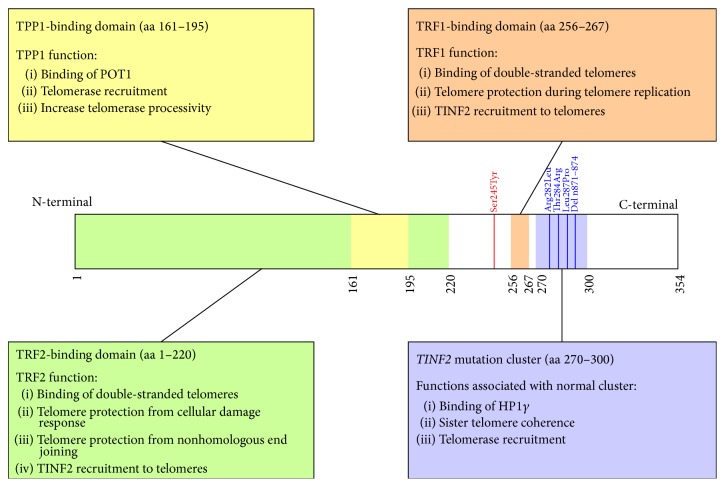
*TINF2* gene overview visualizing the sequence of protein domains and describing the interactions of the TINF2 protein in the shelterin complex. TINF2 mediates the formation of the shelterin complex by binding to the TRF1, TRF2, and TPP1 proteins. Numbers along the lower side of the* TINF2* gene denote encoded amino acid positions. The known binding domains of the TINF2 interaction partners TRF1, TRF2, and TPP1 are indicated in orange, green, and yellow, respectively [15, 16]. TINF2 interacting protein functions are annotated in boxes. TRF1 protein function is based on [17, 18]. TRF2 protein function is based on [17]. TPP1 protein function is based on [17, 19]. TINF2 mutation cluster function is based on [20, 21]. Numbers along the upper side of the* TINF2* gene indicate amino acid positions of* TINF2* mutations in patients with pulmonary fibrosis. The Ser245Tyr mutation location is shown in red. The* TINF2* DC mutation cluster is indicated in purple [22]. Blue lines indicate* TINF2* mutations found in patients with pulmonary fibrosis at amino acids 282 [23], 284 [13], and 287 [24] and nucleotides 871–874 deletion [25]. aa = amino acid.

This case is interesting for multiple reasons. First, this case represents another association between* TINF2* mutations and pulmonary fibrosis. Previously,* TINF2* mutations were found in three DC patients that developed pulmonary fibrosis [23–25] and in a family with pulmonary fibrosis, infertility, and short telomeres [13] (Figure 2). In the literature, five persons with the same heterozygous Ser245Tyr mutation as our patient have been described. These mutation carriers include a 50-year-old male with aplastic anemia [26], a 7-year-old girl with abnormal skin pigmentation and bone marrow failure and her asymptomatic 35-year-old mother [27], and a 14-year-old boy with aplastic anemia and his asymptomatic father [28]. None of these patients had pulmonary fibrosis.

Secondly, the nature of the Ser245Tyr mutation is of interest. The TINF2 protein has multiple roles in telomere maintenance, by way of the binding of TINF2 to several other telomere maintenance proteins (Figure 2), thereby forming the shelterin complex. The majority of the* TINF2* mutations found in telomere syndrome patients lie in a cluster outside the binding regions of TINF2 to shelterin proteins TRF1, TRF2, and TPP1 and do not seem to influence the interaction of TINF2 with these proteins [20, 22]. It has been found that the mutations in this cluster impair the binding of heterochromatin protein 1 gamma (HP1*γ*) to TINF2 [21]. The TINF2-HP1*γ* interaction is involved in sister telomere cohesion during cell division, which is thought to be required for adequate telomerase functioning [21]. A recent study suggests that telomere shortening due to mutations in the* TINF2* mutation cluster is caused by impaired telomerase recruitment to telomeres rather than impaired telomerase functionality [20].

The Ser245Tyr mutation lies outside of this mutation cluster and also outside of the binding regions of TINF2 to TRF1, TRF2, and TPP1. The function of the 245th amino acid of TINF2 is not known at the present, as are the biochemical consequences of the serine to tyrosine substitution at this location. All reported patients with the Ser245Tyr mutation described, including our patient, have normal telomere length. This led the authors of one study to state that this mutation is nonpathogenic [27]. However, the mutation has been associated with a telomere syndrome phenotype in three patients (four when our patient is included), and therefore others consider the Ser245Tyr mutation to be pathogenic, but associated with a milder telomere syndrome phenotype compared to other* TINF2* mutations [28].

In support of the latter position, Sorting Intolerant from Tolerant (SIFT) analysis [29] predicted this mutation to be deleterious (performed at http://sift.jcvi.org/ using default settings; SIFT value 0.00 (version 1.03, reference sequence: NP_001092744.1)). PolyPhen-2 prediction [30] reported that this mutation is possibly damaging (performed at http://genetics.bwh.harvard.edu/pph2/ using default settings; value 0.907 (version 2.2, reference sequence: NP_001092744.1)). Furthermore, we screened 100 self-reported healthy hospital employees, 125 lung transplantation donors, and 63 self-reported healthy other controls, none of whom were found to carry the* TINF2* Ser245Tyr mutation. In addition the minor allele frequency for the mutation in the 1000 genome project [31] is 0.001 (at http://browser.1000genomes.org/, data from Ensembl release 68), indicating very low population frequency.

The fact that our patient, as well as other reported Ser245Tyr mutation carriers, had normal telomere length in the blood compartment does not fit within the model of critically short telomeres that lead to telomere syndrome manifestations. However, we believe that this view of telomere syndrome pathogenesis is incomplete. It has been shown in mice that conditional deletion of* Trf1* or* Trf2* in type 2 alveolar epithelial cells leads to a significant telomere damage response, cellular senescence, and a pulmonary fibrosis phenotype, while maintaining normal telomere length [5, 32]. In addition, in patients with severe telomere syndrome phenotypes, some show very short telomeres in the blood cell compartment, while others show a normal telomere length in the presence of a severe telomere damage response [33]. This points to a model where telomere dysfunction, and not short telomere length per se, is the ultimate cause of a telomere syndrome phenotype. Short telomeres are no prerequisite for telomere dysfunction, and the Ser245Tyr mutation in our patient might influence telomere protection in a manner that is not presently known.

Thirdly, this case draws attention to late-onset hypogammaglobulinemia as a manifestation of telomere syndromes. It is highly likely that our patient developed hypogammaglobulinemia later in life, as she had no history of recurrent infections. Immunodeficiency is commonly seen in DC and can even precede other bone marrow manifestations [34]. The working diagnosis of CVID could not be confirmed in accordance with current diagnostic criteria (available at http://esid.org/Working-Parties/Registry/Diagnosis-criteria/) due to a lack of data on our patients' vaccination responses and isohemagglutinin titer levels. To our knowledge, the association of IPF and CVID has never been reported.

It cannot be definitively stated whether the pulmonary fibrosis in our patient is an independent manifestation of a telomere syndrome, is related to the patients' hypogammaglobulinemia, or is a case of idiopathic pulmonary fibrosis in the strictest sense of the word. There were no radiological or pathological features suggestive of bronchiectasis or granulomatous-lymphocytic interstitial lung disease (GLILD), which are typical pulmonary complications of CVID [35]. When combining this with the (probably) damaging nature of the Ser245Tyr* TINF2* gene mutation, we find it the most plausible that our patients' pulmonary fibrosis was an independent manifestation of a telomere syndrome. Because only one case is presented here, conclusions with regard to the pathogenetic origin of the disease in our patient have to be drawn with caution. The association between the Ser245Tyr* TINF2* gene mutation, pulmonary fibrosis, and hypogammaglobulinemia needs to be validated in other cohorts.

In summary, we identified a heterozygous Ser245Tyr mutation in the* TINF2* gene of a sporadic pulmonary fibrosis patient. This case represents another association between* TINF2* mutations and pulmonary fibrosis. Furthermore, this case illustrates the importance of telomere dysfunction and not telomere length alone in telomere syndromes and draws attention to hypogammaglobulinemia as a manifestation of telomere syndromes.

## Abbreviations

CVID: Common variable immunodeficiency
DC: Dyskeratosis congenita
GLILD: Granulomatous-lymphocytic interstitial lung disease
HP1*γ*: Heterochromatin protein 1 gamma
IPF: Idiopathic pulmonary fibrosis
PPFE: Pleuroparenchymal fibroelastosis
*TINF2*: TRFF1-Interacting Nuclear Factor 2
UIP: Usual interstitial pneumonia.
